# Bioconversion of Beet Molasses to Alpha-Galactosidase and Ethanol

**DOI:** 10.3389/fmicb.2019.00405

**Published:** 2019-03-07

**Authors:** María-Efigenia Álvarez-Cao, María-Esperanza Cerdán, María-Isabel González-Siso, Manuel Becerra

**Affiliations:** Grupo EXPRELA, Centro de Investigacións Científicas Avanzadas (CICA), Facultade de Ciencias, Universidade da Coruña, A Coruña, Spain

**Keywords:** alpha-galactosidase, invertase, *Saccharomyces cerevisiae*, beet molasses, bioconversion, ethanol

## Abstract

Molasses are sub-products of the sugar industry, rich in sucrose and containing other sugars like raffinose, glucose, and fructose. Alpha-galactosidases (EC. 3.2.1.22) catalyze the hydrolysis of alpha-(1,6) bonds of galactose residues in galacto-oligosaccharides (melibiose, raffinose, and stachyose) and complex galactomannans. Alpha-galactosidases have important applications, mainly in the food industry but also in the pharmaceutical and bioenergy sectors. However, the cost of the enzyme limits the profitability of most of these applications. The use of cheap sub-products, such as molasses, as substrates for production of alpha-galactosidases, reduces the cost of the enzymes and contributes to the circular economy. Alpha-galactosidase is a specially indicated bioproduct since, at the same time, it allows to use the raffinose present in molasses. This work describes the development of a two-step system for the valuation of beet molasses, based on their use as substrate for alpha-galactosidase and bioethanol production by *Saccharomyces cerevisiae*. Since this yeast secretes high amounts of invertase, to avoid congest the secretory route and to facilitate alpha-galactosidase purification from the culture medium, a mutant in the *SUC2* gene (encoding invertase) was constructed. After a statistical optimization of culture conditions, this mutant yielded a very high rate of molasses bioconversion to alpha-galactosidase. In the second step, the *SUC2* wild type yeast strain fermented the remaining sucrose to ethanol. A procedure to recycle the yeast biomass, by using it as nitrogen source to supplement molasses, was also developed.

## Introduction

Molasses are viscous dark sub-products resulting from the sugar-making industry; they are rich in sucrose and contain lower amounts of other sugars like raffinose, glucose, and fructose. Due to the high sucrose content (about 50% by dry weight), at present molasses serve mostly as substrate for the industrial production of bioethanol by the yeast *Saccharomyces cerevisiae* (Akbas and Stark, 2016). The use of food industry wastes as sustainable substrates for the microbial synthesis of other biotechnological products besides ethanol, such as enzymes and other active ingredients, is increasingly gaining field in the context of the circular economy (da Silva, 2016). From this point of view, molasses provides a good carbon source for microbial cell growth (Santos et al., 2010), and the yeast biomass itself, generated in the bioprocess, may be used after a simple treatment (Ferreira et al., 2010) as source of nitrogen and B-group vitamins to complement molasses.

*Saccharomyces cerevisiae* is undoubtedly the best performing microorganism for molasses fermentation to bioethanol (Akbas et al., 2014). This yeast is also a widely employed host for heterologous protein production and secretion (Nielsen, 2013). However, there are no reports hitherto on the use of molasses as substrate with the aim of heterologous protein secretion by *S. cerevisiae*.

Alpha-galactosidases (EC. 3.2.1.22) catalyze the hydrolysis of alpha-(1,6) bonds of galactose residues in galacto-oligosaccharides (melibiose, raffinose, and stachyose) and complex galactomannans. Some alpha-galactosidases can also synthesize oligosaccharides by transglycosylation reactions under substrate supersaturation conditions (Spangenberg et al., 2000). Indeed alpha-galactosidases show a great variety of uses, mainly in the food industry such us improvement of separation of sucrose from beet (Linden, 1982), reduction of the content of non-digestible oligosaccharides of legume-derived food products (Katrolia et al., 2012), obtention of the low-calorie sweetener tagatose (Kim et al., 2009), improvement of rheological properties of galactomannans (Dey et al., 1993), and synthesis of prebiotics (Dai et al., 2018), but also in other sectors like pharmaceutical (Arends et al., 2017) and bioenergy (Rodrigues-Dutra et al., 2017). *S. cerevisiae* alpha-galactosidase (ScAGal), encoded by the *MEL1* gene (GeneBank X03102), is a highly glycosylated 471-amino acid extracellular protein, and the crystal structures of the complexes with melibiose and raffinose have been reported (Fernández-Leiro et al., 2010).

The cost of the enzyme actually limits the profitability of most of the above cited applications. The use of cheap sub-products, such as molasses and whey, as substrates for production of ScAGal, was previously reported and might favor the economy of the processes (Álvarez-Cao et al., 2018). The aim of this work is to develop a system for the valuation of beet molasses, based on their use as substrate for ScAGal and bioethanol production by *S. cerevisiae* in two-steps. A first step using an invertase-deficient mutant (transformed with a ScAGal over-expressing plasmid), which not only favors the secretion-purification of the enzyme due to the absence of invertase (Liljeström et al., 1991), but also increases the yield of ScAGal production, because the mutant uses the sugars present at low concentration in the molasses but not sucrose, and then the metabolism is preferentially respiratory under aerobic conditions. In the second step, the invertase wild-type strain is used to convert the sucrose to ethanol under yet established fermentation conditions.

With the engineered strains and two-step system here developed, a high rate of molasses bioconversion to alpha-galactosidase was obtained (culture conditions were statistically optimized), and sucrose was efficiently converted to ethanol. A procedure to recycle the yeast biomass, by using it as nitrogen source to supplement molasses, was also developed.

## Materials and Methods

### Microorganisms, Expression Vectors, and Culture Media

*Saccharomyces cerevisiae* BJ3505 [*pep4::HIS3, prb*-Δ*1.6R HIS3, lys2-208, trp1*-Δ*101, ura 3-52, gal2, can1*] (Eastman Kodak Company) was selected to construct a mutant depleted of invertase activity to be used as host for heterologous protein expression. *Escherichia coli* XL1-Blue [*recA1 endA1 gyrA96 thi-1 hsdR17 supE44 relA1 lac [F'proAB lacIqZDM15 Tn10 (Tetr)]*] (Stratagene Cloning Systems) was employed for standard DNA recombinant techniques (Ausubel et al., 2003).

The plasmids YEp*MEL1* [ampr ori 2 μ *MEL1 TRP1*] and YEp*MEL1*His [ampr ori 2 μ *MEL1*His *TRP1*] (Fernández-Leiro, 2011), bearing the *MEL1* gene that encodes ScAGal, were chosen as expression vectors. The vector YEpFLAG-1 (Eastman Kodak Company) was used as control of the expression system.

LB [1% (w/v) tryptone, 0.5% (w/v) yeast extract, 0.5% (w/v) NaCl], and YPD [1% (w/v) yeast extract, 2% (w/v) peptone, 2% (w/v) glucose] were used as culture media for growth and maintenance of bacteria and yeasts, respectively. LB was supplemented with 100 mg/L ampicillin (Sigma Aldrich) for the propagation of plasmids in bacteria. A complete medium without uracil (CM-Ura) or tryptophan (CM-Trp) (Zitomer and Hall, 1976) was used for selection in *S. cerevisiae*. Two percent (w/v) bacteriological agar was added to solid media. A modified YPHSM medium and beet molasses (provided by AB Azucarera Iberia, Spain) based media were used for ScAGal production. Molasses were diluted in distilled water ratio 1:1 (v/v), to facilitate handling, and centrifuged at 10,000 rpm for 15 min to remove solid impurities. Ampicillin was filtered through 0.22 μm membrane (Sartorius AG) and the rest of media components were sterilized by autoclave at 121°C for 20 min. The composition of the media used in this work is shown in Table 1.

**Table 1:** Summary of the strategy of study of media and culture conditions on the production of ScAGal.

	**Media**	**Inoculum**	**Strain/s**
1. Expression system selection:	Modified YPHSM: 1% (w/v) yeast extract, 8% (w/v) peptone, 3% (w/v) glycerol, 2% (w/v) glucose	OD_600_ = 0.5	BJ3505Δ*suc2*/YEp*MEL1* BJ3505Δ*suc2*/YEp*MEL1*His BJ3505/YEp*MEL1* BJ3505/YEp*MEL1*His
2. Evaluation beet-molasses media:	YR: 1% (w/v) yeast extract, 8% (v/v) beet molasses PR: 2% (w/v) peptone, 8% (v/v) beet molasses	OD_600_ = 2	BJ3505Δ*suc2*/YEp*MEL1* BJ3505Δ*suc2*/YEpFLAG-1 BJ3505/YEp*MEL1* BJ3505Δ*suc2*/YEpFLAG-1
3. Recycling of biomass:	YR: 1% (w/v) yeast extract, 8% (v/v) beet molasses YR_aut_: 0.5% (v/v) autolyzed biomass, 8% (v/v) beet molasses	OD_600_ = 4	BJ3505Δ*suc2*/YEp*MEL1*
4. Growth kinetic and conversion substrates to products:	YR: 1% (w/v) yeast extract, 8% (v/v) beet molasses	OD_600_ = 4	BJ3505Δ*suc2*/YEp*MEL1* BJ3505/YEp*MEL1*

### Construction of an Invertase Defective Mutant

**Figure 1:**
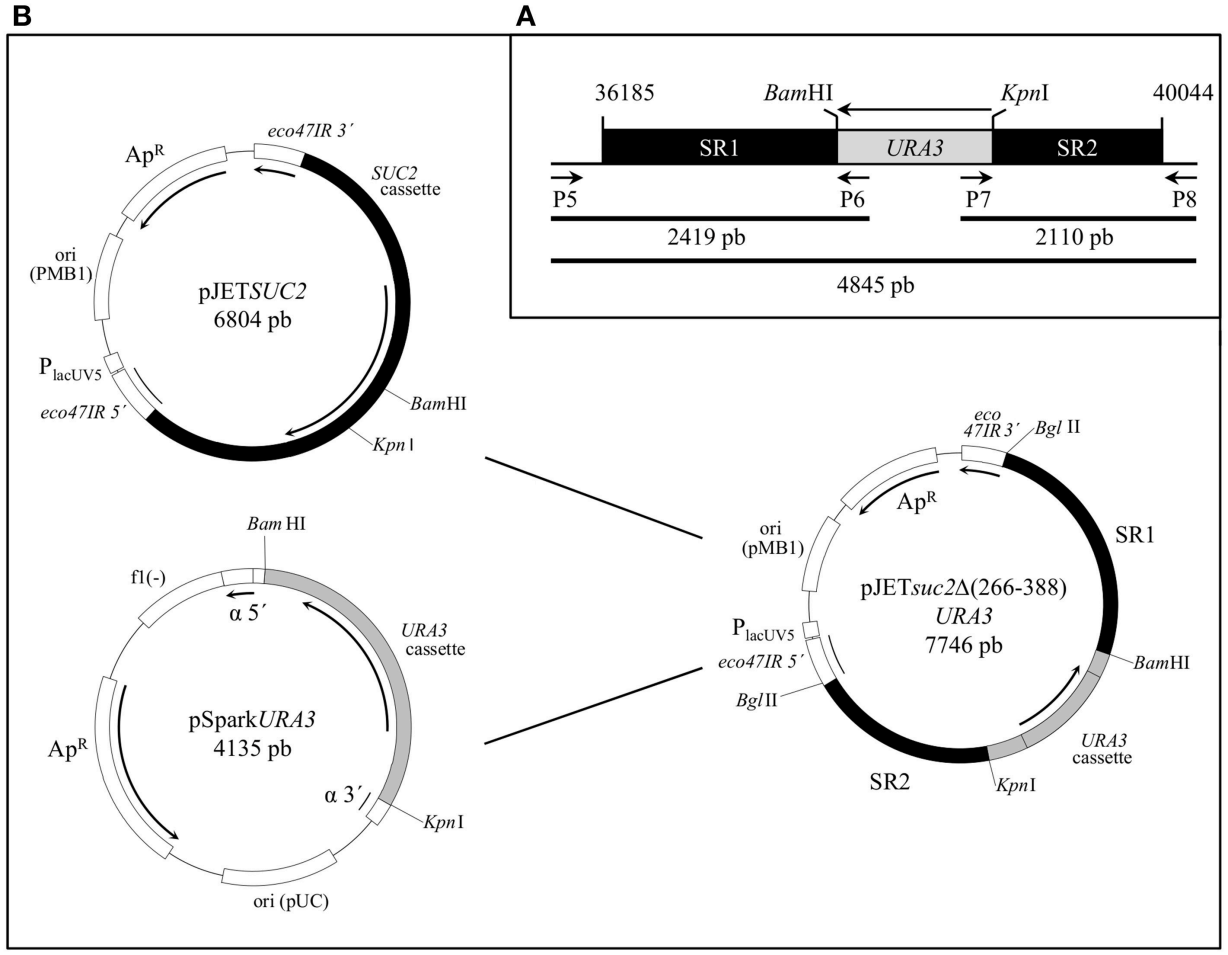
Construction of *S. cerevisiae* strain BJ3505Δ*suc2*. **(A)** The sequence [36,185–40,044] bp of chromosome 9 of *S. cerevisiae* (Gene ID: 854644; NC_001141.2) containing the *SUC2* ORF was chosen to carry out the *suc2*Δ(266–388) deletion by replacement with the *URA3* cassette between sites *Bam*HI and *Kpn*I. SR1 and SR2 are the chromosomal recombination sequences and the primers pairs P5-P6, P7-P8, and P5-P8 are used to obtain the fragments 2,419, 2,110, and 4,845 bp, respectively to check the chromosomal integration. **(B)** Physical maps of the plasmids constructed to generate strain BJ3505Δ*suc2*: the *URA3* cassette from pSpark*URA3* was cloned between the *Bam*HI-*Kpn*I sites of pJET*SUC2* to obtain pJETs*uc2*Δ(266–388)*URA3*, which was digested with *Bgl*II to release the s*uc2*Δ(266–388)*URA3* cassette that was integrated through SR1 and SR2 sequences into genome of the origin strain.

The *SUC2* gene was deleted in the *S. cerevisiae* strain BJ3505 using the integrative cassette *suc2*Δ(266-388)*URA3* that contains the *suc2*Δ(266-388) deletion and the *URA3* gene as selection marker (Figure 1). All oligonucleotides used as primers in this work are showed in Table S1.

#### Construction of SUC2 and URA3 Cassettes

The ORF of the *SUC2* gene was identified by means of the tool *BLAST* at *GeneBank* (http://www.ncbi.nlm.nih.gov/) and the homology among the different *S. cerevisiae* strains in the database. The nucleotide sequence [36185–40044] in the chromosome 9 of *S. cerevisiae* S288c (*Gene ID*: 854644; NC_001141.2) was used and the design of the integrative cassette is showed in Figure 1A. A region longer than 1 Kb upstream and downstream of the *SUC2* ORF was used to compete with the marker *ura3-52* of the strain BJ3505. The fragment of 3,831 bp length corresponding to the *SUC2* cassette was amplified with the primer pairs P1–P2 (Table S1) from genomic DNA of the strain BJ3505 as template, by the protocol described in Hoffman (2001). A fragment of 1,308 bp flanked by the restriction sites *Bam*HI and *Kpn*I was amplified from the vector YEplac195 (Gietz and Sugino, 1988) using the primer pairs P3–P4 (Table S1) to obtain the cassette *URA3* with its promoter and terminator regions. PCRs were performed using Phusion High-Fidelity DNA Polymerase (Thermo Fisher Scientific).

#### Construction of Plasmids pJETSUC2, pSparkURA3, and pJETsuc2Δ(266–388)URA3

The PCR products obtained from amplification of *SUC2* and *URA3* cassettes were cloned into vectors pJET1.2/*blunt* (CloneJET PCR Cloning Kit, Thermo Fisher Scientific) and pSpark IV (pSpark DNA Cloning System, Canvax Biotech) respectively, following the protocol recommended by each supplier, to create the plasmids pJET*SUC2* and pSpark*URA3*. The *Bam*HI-*Kpn*I fragment of 1,308 bp from pSpark*URA3* was cloned between the *Bam*HI-*Kpn*I sites of pJET*SUC2* into chemically competent XL1-Blue cells to construct the plasmid pJET*suc2*Δ(266–388)*URA3* with the recombination sequences SR1 (1,962 bp) and SR2 (1,503 bp) that allowed the chromosomal integration (Figure 1B). All the plasmids generated in this work were propagated and extracted from the bacterial cells using GeneJET Plasmid Miniprep Kit (Thermo Fisher Scientific). The inserts were identified by PCR of transformant colonies using DreamTaq polymerase (Thermo Fisher Scientific) and restriction analysis, and correct sequences confirmed by sequencing (Servizos de Apoio á Investigación, Universidade da Coruña).

#### Integration of the suc2Δ(266–388)URA3 Cassette in the BJ3505 Genome

**Figure 2:**
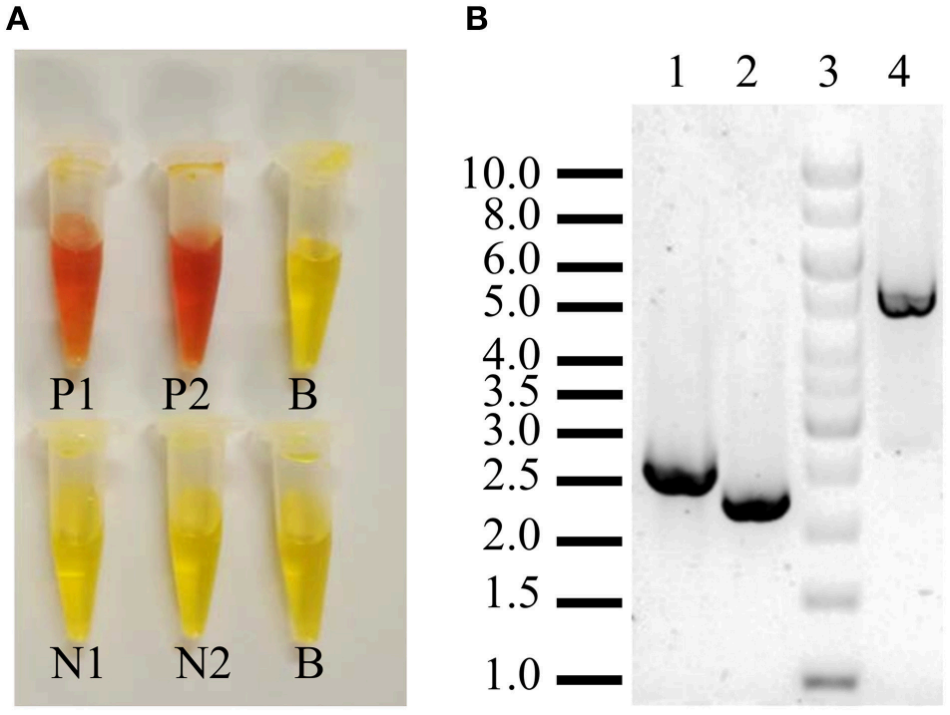
Verification of *S. cerevisiae* strain BJ3505Δ*suc2*. **(A)** Negative (yellow color) and positive (orange color) functional assay of extracellular (1) and intracellular invertase activity (2) as described in section Analytical Methods. N, strain BJ3505Δ*suc2*; P, strain BJ3505 (parental); B, blank. **(B)** PCR analysis of the “upstream” (2,419 bp, lane 1), “downstream” (2,110 bp, lane2), and between them (4,845 bp, lane 4) of the *SUC2* gene that codes for the invertase. Lane 3, GeneRuler 1Kb DNA Ladder (Thermo Fisher Scientific).

The plasmid pJET*suc2*Δ(266–388)*URA3* was digested with *Bgl*II to separate the cassette *suc2*Δ(266–388)*URA3* and further cloning in BJ3505 competent cells transformed by the lithium acetate method (Ito et al., 1983). Chromosomal integration succeeded by two homologous recombination points provided by SR1 and SR2 sequences. Recombinants were selected by growth in CM-Ura and the invertase-deficient strain, named BJ3505Δ*Suc2*, was verified by functional and PCR analysis using DreamTaq polymerase (Thermo Fisher Scientific) (Figure 2).

### Transformation and Inoculum Preparation for ScAGal Production Cultures

The strains BJ3505Δ*Suc2* and BJ3505 were transformed with plasmids YEpFLAG-1, YEp*MEL1*, and YEp*MEL1*His, by a modification of the lithium acetate method (Chen et al., 1992). Transformants were selected on solid CM-Trp after 48 h at 30°C. A single colony was taken to prepare a liquid CM-Trp pre-culture up to an optical density at 600 nm (OD_600_) of 5 (stationary phase) at 30°C and 250 rpm for 72 h, which was used to inoculate up to units OD_600_ corresponding to each production culture tested, as described above.

### Influence of Media and Culture Conditions on ScAGal Production

Preliminary studies on cell growth determined by optical density at 600 nm (Ausubel et al., 2003) and ScAGal production from strains BJ3505Δsuc2 and BJ3505 were performed with PR, YR, and YR_aut_ media (Table 1). Beet molasses were added at the desired concentration, determined as total sugars percentage, and supplemented with yeast extract or peptone to provide nitrogen source.

Yeast extract coming from biomass of ended-cultures was prepared according to a modification of the autolysis method described by Belem et al. (1997). Yeast cells were centrifuged (5,000 rpm, 15 min, 4°C), resuspended 1:2 (w/v) in 0.1 M KH_2_PO_4_ (pH 6.5), and autolyzed at 50°C and 150 rpm for 30 h. Supernatant was recovered by centrifugation (10,000 rpm, 15 min), cell debris was resuspended 1:10 in the same buffer and disintegrated by sonication (Sonics Vibra cell) for 10 min at 7 s intervals to facilitate the release of the remaining soluble cellular material in a second supernatant recovered by centrifugation. Both supernatants were mixed and added at 0.5% (v/v) to supplement the culture media. The RNA released from the final autolysate was analyzed by 1.5% (w/v) agarose gel electrophoresis using 1% (w/v) commercial yeast extract as control (Figure S1A).

Cultures were inoculated, from the pre-cultures of transformed strains described above, by triplicate in flasks containing 20% volume of medium, and incubated at 30°C and 250 rpm. A modified YPHSM medium (Table 1) inoculated to obtain an initial OD_600_ of 0.5 was used to statistically compare the expression systems YEp*MEL1* and YEp*MEL1*His by analysis of variance (ANOVA) with 95% confidence intervals. Profiles of sugar consumption and products formation in the molasses-based media YR and PR were initially determined from cultures inoculated at OD_600_ of 2. Cultures in YR and YR_aut_ media inoculated at OD_600_ of 4 were used to compare the assimilation of the carbon source with commercial yeast extract and autolyzed biomass, respectively. Growth kinetics and substrate to products conversion was evaluated from YR cultures inoculated at OD_600_ of 4. In this case, when the consumption of sugar was negligible, half of each culture volume was separated while the other half was maintained in the same conditions. Under sterile conditions, the volume separated of each culture was centrifuged (5,000 rpm, 10 min, 4°C), the supernatant was refreshed with 1% (w/v) yeast extract and inoculated at the same initial OD_600_ with cells coming from the yeast pellet. Cultures were stopped after 84 h and the last step was repeated but only with the BJ3505Δ*suc2* cultures and using as inoculum the BJ3505 at the same initial OD_600._ Samples were taken at different incubation times to determine biomass, residual sugars, ethanol, and enzyme activities (invertase and alpha-galactosidase). A summary of the strategy of study of media and culture conditions is shown in Table 1.

### Microscopy

An optical microscope (Nikon Eclipse 50) was used to observe directly, without fixation, the cells from the collected culture samples. For observation under transmission electron microscopy (TEM) (Servizos de Apoio á Investigación, Universidade da Coruña), the cells were fixed in 100 mM sodium cacodylate buffer pH 7.2 as described in Bauer et al. (2001).

### Optimization of ScAGal Production by the Surface Response Methodology

Statistical optimization of beet molasses based cultures for ScAGal production was performed using the response surface methodology (RSM) with the strain BJ3505Δ*suc2*/YEp*MEL1*. The experimental factors selected as independent variables were 4: concentration of molasses and yeast extract, inoculum size, and culture time. The dependent variable or response was extracellular alpha-galactosidase activity. A central composite design (CCD) was applied to study the effects and interactions among the four factors at five different levels. Their coded values were –α, −1, 0, +1, +α, being α = 2^k/4^, *k* the number of independent variables and 0 the central point. Table 2 shows the levels and real values of the factors, calculated according to the equation described by de Faria et al. (2013). The RS was adjusted to a polynomic second order equation that correlates the measured response with the independent variables (Dilipkumar et al., 2014), and the optimum value of the CCD was obtained solving the regression equation and analyzing the surface response contour plots. An ANOVA with 95% confidence intervals was performed to determine the significance of the effects.

**Table 2:** Experimental domain and codification of the independent variables in the CCD to the optimization of the ScAGal production by BJ3505Δs*uc2*/YEp*MEL1*.

**Real values**	**Coded values**^**a**^				
	**−2**	**−1**	**0**	**1**	**2**
Beet molasses (%), X_1_	11	12.5	14	15.5	17
Yeast extract (%), X_2_	1	1.5	2	2.5	3
Inoculum size (OD_600_), X_3_	0.5	2.5	4.5	6.5	8.5
Culture time (h), X_4_	36	60	84	108	132

a *x_i_ = (X_i_-X_0_)/ΔX_i_, i = 1, 2, 3, 4; where x_i_ and X_i_ are the coded and real values of the independent variable i, X_0_ is the real value of the independent variable i at the central point, and ΔX_i_ is the step change value*.

### Bioreactor Operation

Bioreactor cultures were run in a Biostat-MD (Braun-Biotech) 2 L fermentor, 1 L working volume, with pH control to evaluate its effect on ScAGal production. For this, the fermentations were carried out without and with adjustment to pH 6 during the course of the culture. Medium composition and inoculum size were selected from the results of the CCD described above. Bioreactor was sterilized at 121°C for 30 min, and then aseptically inoculated and supplemented with 300 mg/L ampicillin. Culture conditions were 30°C, 2 L/min air flow and 300 rpm, for 120 h (Ausubel et al., 2003). Sterilized 1 M NaOH or 1 M HCl were added as needed to adjust pH. Samples were taken at regular time intervals to determine yeast biomass, extracellular alpha-galactosidase activity, and conversion of substrates into secondary metabolites. Plasmid stability was determined as percentage of viable Trp^+^ colonies, after seeding diluted samples of the culture on Petri dishes with CM and CM-Trp and counting colony forming units by the following equation,

$$\begin{array}{l} \text{Percentage plasmid stability }\left(\%\right)=\\ \;\frac{\text{Colony Forming Units }\left\{\text{CM}-\text{Trp}\right\}\;}{\text{Colony Forming Units }\left\{\text{CM}\right\}}\times 100 \end{array}$$

### Analytical Methods

#### Molasses Sugar and Protein Content

The sugars present in the diluted molasses used to prepare the culture media were identified and quantified by High-Performance Liquid Chromatography (HPLC) using an external standard formed by known concentrations of raffinose, sucrose, glucose, and fructose ranging 4–0.06 mg/mL. An estimate of the nitrogen fraction in the diluted molasses according to the protein content was measured by the Bradford's method using DC Protein Assay Kit (Bio Rad) and bovine serum albumin as standard.

#### Biomass, Residual Sugars, and Ethanol Determination

Cell growth in shaken flasks was measured as units of OD_600_ with an UV-Visible espectrophotometer (Biospectrometer Kinetic Eppendorf) to express the biomass generated. Cell-free medium was used to measure ethanol, with the enzymatic assay Ethanol UV method (NZYTtech), and residual sugar. The latter was quantified indirectly using the DNS method (Miller, 1959) by means of the analysis of reducing sugars content in the samples before and after hydrolysis at 95°C for 5 min with 18.5% (w/v) HCl followed by neutralization with 25% (w/v) NaOH. For cultures in bioreactor, biomass was quantified as dry weight from 5 mL samples, after being centrifuged at 5,000 rpm for 5 min, washed with distilled water, centrifuged again, and dried at 105°C until constant weight. The supernatant was used to measure sugar consumption, ethanol, and other metabolites formation by HPLC using an external standard composed by known concentrations of raffinose, sucrose, galactose, glucose, fructose, glycerol, and ethanol ranging 4–0.06 mg/mL.

#### HPLC Analysis

Sugar Pack Waters (6.5 × 300 mm) column and Refractive Index Detector (Cienytech) were used. Samples were previously clarified with cartridges HyperSep Silica SPE Column (Fisher Scientific) to avoid interferences due to pigments and other impurities coming from molasses. Column temperature 80°C, detector temperature 37°C, and sensitivity 32 were the running conditions using as mobile phase 100 μM EDTA-Calcium (Sigma Aldrich) at flow rate of 0.5 mL/min. Eluted compounds were identified and quantified using sorbitol (1 mg/mL) as internal standard, since it does not interfere with the retention times of the compounds under study (Xu et al., 2015).

#### Enzyme Activity Assay

Extracellular alpha-galactosidase activity was measured by the method of Ryan et al. (1998), incubating the samples with a 10 mM solution of the synthetic substrate *p*-nitrophenyl-α-galactose (Sigma Aldrich) in McIlvaine buffer (pH 4) at 40°C. At two consecutive time intervals, the reaction was stopped with 0.5 M Na_2_CO_3_ and released *p*-nitrophenol was measured at 400 nm (molar extinction coefficient: 18.20 mmol^−1^.cm^−1^). Invertase activity, extracellular, and intracellular was measured from supernatant and sediment of culture samples, respectively, after permeabilization with 15% (v/v) chloroform and 0.01% (w/v) SDS. The samples were incubated with 100 mM sucrose in 50 mM acetate buffer (pH 5) for 10 min at 40°C, and reducing sugars were determined by the DNS method (Miller, 1959). One enzyme unit (U) is defined as the amount of enzyme that releases one μmol of product per minute under assay conditions.

#### Statistical Analysis

Statistical data treatment was done using the StatGraphics Centurion XVI package and graphics construction was performed with the program SigmaPlot 12.0. Significance of data was evaluated with Student's *t*-test and results were considered significant for *p* ≤ 0.05.

## Results

### Construction of an Invertase Defective Mutant

The strain BJ3505Δ*suc2* was constructed by the procedure described in the Materials and Methods section. The correctness of the mutation was verified by functional measurement of invertase activity that was fully depleted in the mutant (Figure 2A) and by PCR analysis (Figure 2B, see also Figure 1A) that showed bands of the expected size in function of the chromosomal integration.

### Approach to a Medium for ScAGal Production Based on Beet Molasses

**Figure 3:**
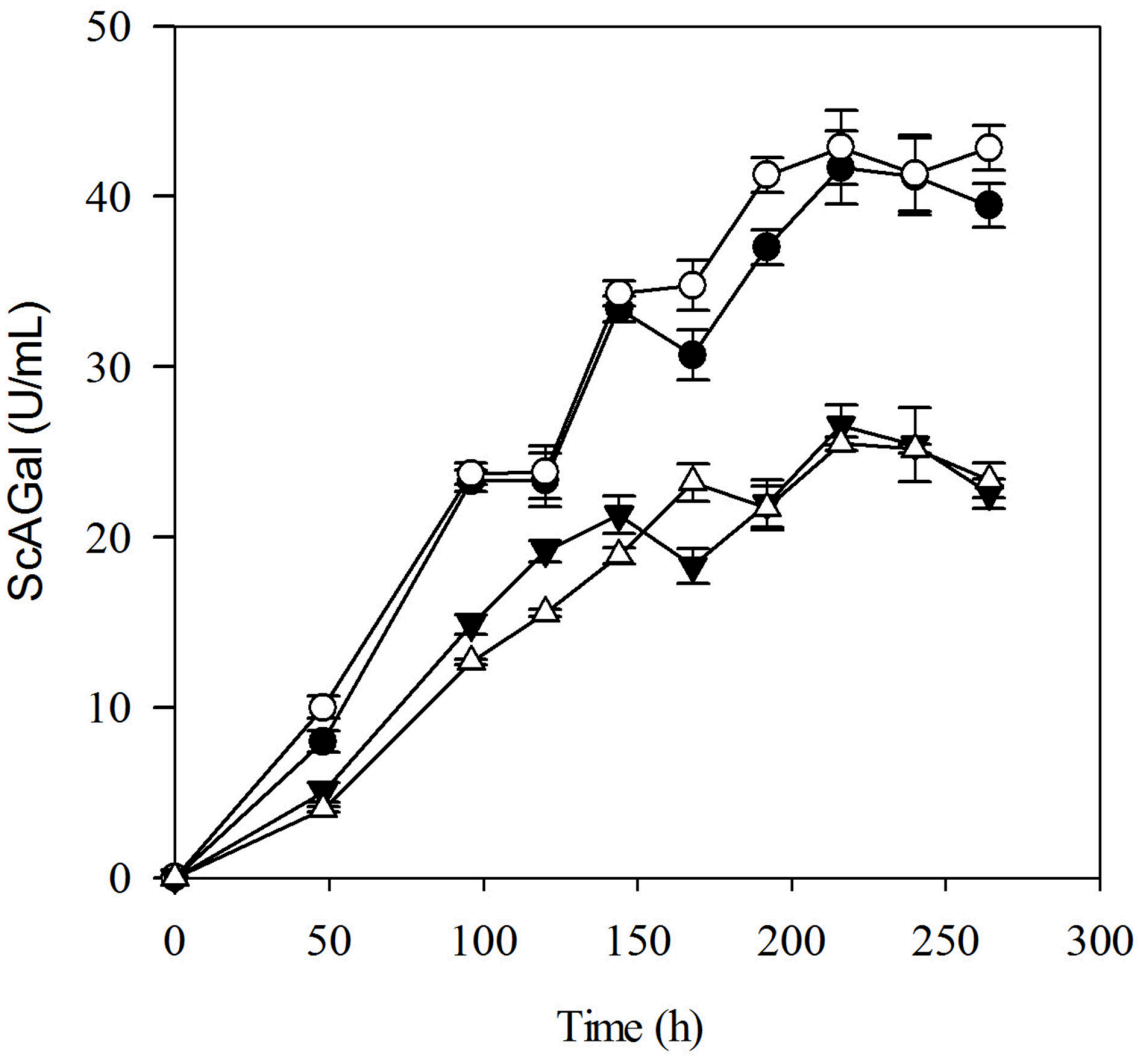
Time course of extracellular alpha-galactosidase activity using a conventional culture medium. Cultures in the YPHSM medium were inoculated to an OD_600_ of 0.5 from pre-cultures of strains BJ3505Δ*suc2* (empty symbol) and BJ3505 (full symbol) transformed with the plasmids YEp*MEL1* (circle), and YEp*MEL1*His (triangle).

Cultures in YPHSM of the strains BJ3505Δ*suc2* and corresponding wild type BJ3505, transformed with the plasmids YEp*MEL1*His or YEp*MEL1*, with and without affinity purification tag, respectively, were performed to select the best ScAGal expression system. YPHSM is the recommended medium for heterologous protein expression with the YEpFLAG-1 system (Eastman Kodak Company) that was used to construct the plasmids (Fernández-Leiro, 2011). Growth was similar in the four cases (OD_600_ = 90 ± 10; data not shown), and maximum extracellular alpha-galactosidase activity was similar for the two strains but dependent on the plasmids, 43 and 25 U/mL for YEp*MEL1* and YEp*MEL1*His, respectively, after 216 h of incubation (Figure 3). Although the statistical analysis shows that there is not a direct correlation between ScAGal secretion and lack of invertase in glucose (YPHSM) medium (Table S2), the advantage of obtaining alpha-galactosidase preparations not contaminated with invertase is clear. Therefore, BJ3505Δ*suc2*/YEp*MEL1* was selected as an improved ScAGal expression system for the following experiments.

**Figure 4:**
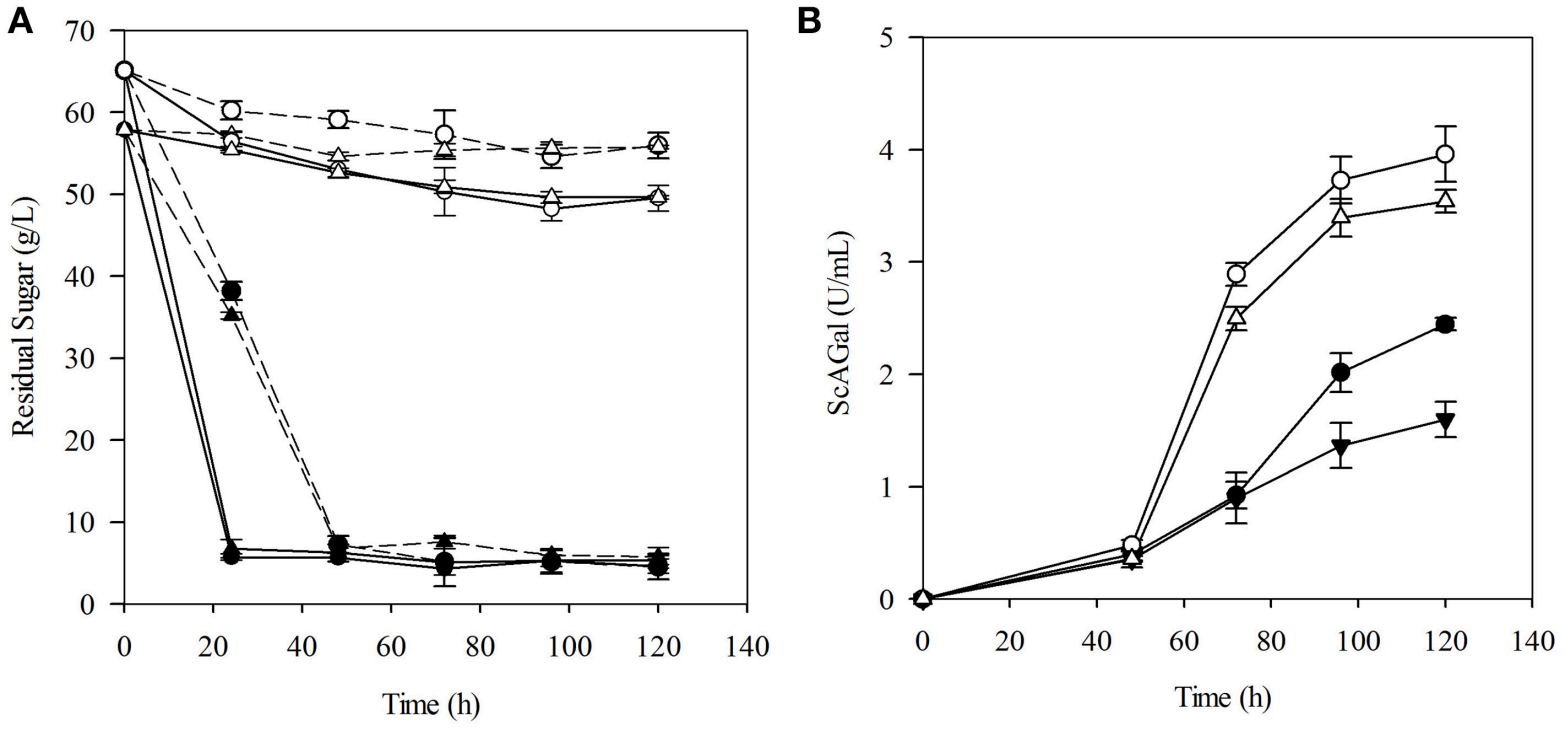
Evaluation of culture media based on beet molasses for ScAGal production. Time course of residual sugar **(A)** and extracellular alpha-galactosidase activity **(B)** by BJ3505Δ*suc2* (empty symbol) and BJ3505 (full symbol) in culture media YR (triangle) and PR (circle). The solid and dashed lines correspond to the strains transformed with the plasmid YEp*MEL1* and YEpFLAG-1, respectively. The media YR and PR were prepared with 8% beet molasses supplemented with 1% yeast extract or 2% peptone and the cultures were inoculated to OD_600_ = 2.

Raw molasses used in this work contained 59.7% sucrose, 2.9% raffinose, 2.4% fructose, 1.2% glucose, and 0.3% nitrogen fraction. Beet molasses are limited in biotin (vitamin B7) and other sources of nitrogen, and yeast biomass can be valuable for using as nitrogen and vitamins supplement for microbial growth (Ferreira et al., 2010). Then, the effects on sugar consumption and ScAGal extracellular production of molasses supplementation with yeast extract and peptone using the YR and PR media, respectively, were assayed and are shown in Figure 4. Results prove that, as expected due to the Δ*suc2* mutation, the strain BJ3505Δ*suc2* uses only 10–20% of the sugars provided by the media, while the strain BJ3505 (with intact *SUC2* gene) uses the 90% at the same culture time. An increase of sugar consumption rate was observed for strains transformed with YEp*MEL1* due to alpha-galactosidase activity (Figure 4A). From Figure 4B, it is inferred that in YR and PR media, BJ3505Δ*suc2*/YEp*MEL1* produces more extracellular ScAGal than BJ3505/YEp*MEL1* does, i.e., indeed invertase depletion favors ScAGal secretion in sucrose-rich medium, and that yeast extract can be used as supplement instead of peptone, since ScAGal production by BJ3505Δ*suc2*/YEp*MEL1* is quite similar with both supplements. Furthermore, the recycling of yeast biomass as molasses supplement was analyzed and it was found that the autolysis treatment provides a source rich in low molecular weight nucleotides, as is well-documented (Belem et al., 1997). Although the results also confirmed very close values of sugar consumption and extracellular ScAGal production (Figure S1B), we continued to use commercial yeast extract for the following experiments, as it is more standard and reproducible among laboratories.

**Figure 5:**
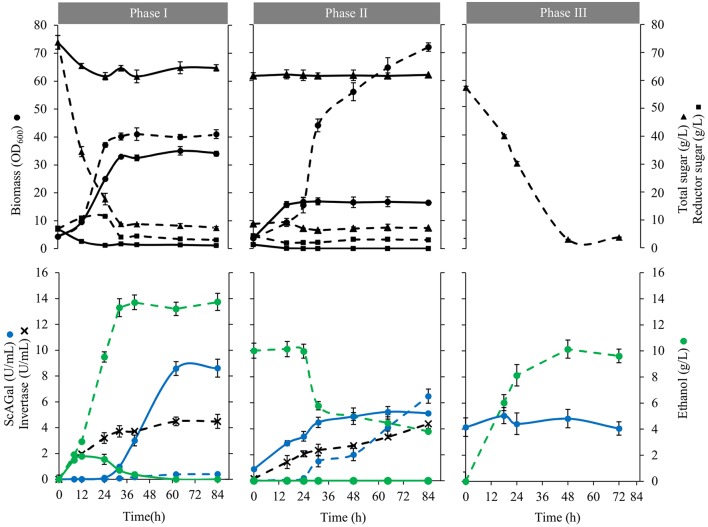
Growth kinetic and conversion of substrates to products of strains BJ3505Δs*uc2*/YEp*MEL1* and BJ3505/YEp*MEL1*. Biomass (circles), total sugar (triangles), reducing sugar (squares), ethanol (green circles), extracellular alpha-galactosidase activity (blue circles), and extracellular invertase activity (blades) of cultures BJ3505Δ*suc2*/YEp*MEL1* (solid line) and BJ3505/YEp*MEL1* (dashed line). The cultures were inoculated in the YR production medium (8% beet molasses, 1% yeast extract) (Phase I). After a period of growth of 40 h, part of each of the cultures was separated and cooled with 1% yeast extract (Phase II) while the rest remained in the same conditions of phase I. The free medium of cells recovered from final cultures of BJ3505Δ*suc2*/YEp*MEL1* was reused as a production medium using strain BJ3505/YEp*MEL1* (Phase III). In each phase the same initial cell density was maintained (OD_600_ = 4).

Figure 5 shows culture profiles of BJ3505Δ*suc2*/YEp*MEL1* and the corresponding wild type BJ3505/YEp*MEL1* in 8% beet molasses supplemented with 1% yeast extract. It is observed that both strains reach similar growth levels but while BJ3505/YEp*MEL1* consumed 88% sugars (native invertase hydrolyzed sucrose) and produced 13 g/L of ethanol, BJ3505Δ*suc2*/YEp*MEL1* consumed 12% sugars producing only 1.8 g/L ethanol that was later metabolized as carbon source (Phase I). After 40 h of incubation, all available sugar was consumed by both strains and a part of the cultures was supplemented again with 1% yeast extract, which caused that BJ3505/YEp*MEL1* shifted to use ethanol as carbon source and to secrete ScAGal, reaching a biomass four-fold higher than BJ3505Δ*suc2*/YEp*MEL1* that continued secreting ScAGal (Phase II). Finally, the cell free medium at the end of the BJ3505Δ*suc2*/YEp*MEL1* culture was inoculated with BJ3505/YEp*MEL1* that converted the sucrose into ethanol (Phase III). So, the metabolism of BJ3505/YEp*MEL1* is fermentative and produces ethanol from sucrose in 8% molasses, even under aerated conditions, known as Crabtree effect (Marques et al., 2015). However, BJ3505Δ*suc2*/YEp*MEL1* shows a respiratory metabolism, probably due to the lower amount of sugars available since this strain cannot use sucrose, and metabolizes the rest of sugars present in molasses producing alpha-galactosidase more efficiently.

**Figure 6:**
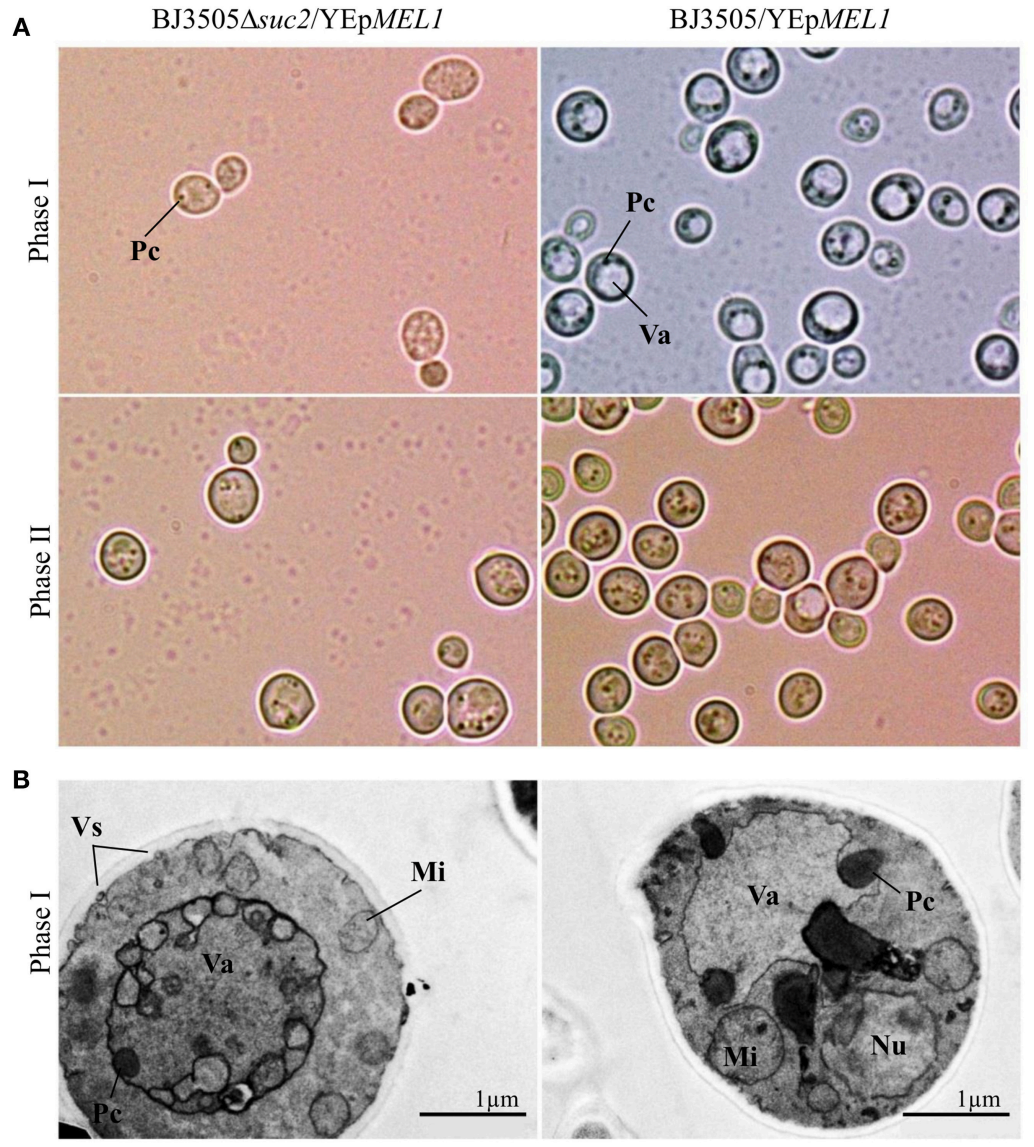
Visualization of cultures of BJ3505Δ*suc2*/YEp*MEL1* and BJ3505/YEp*MEL1* by optical microscopy **(A)** and TEM **(B)**. Va, vacuole; Pc, Cytoplasmic particles; Nu, nucleus; Mi, mitochondria; Vs, secretion vesicles.

Moreover, both strains BJ3505Δ*suc2*/YEp*MEL1* and BJ3505/YEp*MEL1* show morphological differences depending on the culture phase (Figure 6). By optical microscopy, the presence of particles around the vacuole is observed in BJ3505/YEp*MEL1*, while no vacuole is visible in BJ3505Δ*suc2*/YEp*MEL1* and there are fewer particles in the cytoplasm (Phase I, Figure 6A). However, when BJ3505/YEp*MEL1* starts to use ethanol as carbon source, the morphology of both strains is similar (Phase II, Figure 6A). The TEM images show in more detail the intense cytoplasmic activity in BJ3505Δ*suc2*/YEp*MEL1* possibly as a result of the production of heterologous protein compared to the strain of origin (Phase I, Figure 6B).

### Optimization of ScAGal Production by BJ3505Δ*suc2*/YEp*MEL1* From Beet Molasses

The matrix of CCD shown in Table 3 defined a set of 30 experiments, including six replicas of the central point to estimate the experimental error of the production technique. The corresponding results are also shown. Following, the surface response methodology (SRM) was applied to the experimental observed data in order to adjust the response function (extracellular alpha-galactosidase activity) and reveal the simultaneous influence of the four studied variables (molasses concentration, yeast extract concentration, inoculum size, and incubation time) on it. The best condition for ScAGal production corresponded to experiment number 13 that reached an extracellular alpha-galactosidase activity of 17 U/mL. In contrast, the worst condition (experiment 23) showed an activity value as low as 0.001 U/mL.

**Table 3:** Experimental matrix according to the CCD and results observed and predicted by the MSR to the optimization of the ScAGal production by BJ3505Δs*uc2*/YEp*MEL1*.

**Exp no**.	**Coded values**				**Real values**^**a**^				**(U/mL)**^**b**^	
	***x_**1**_***	***x_**2**_***	***x_**3**_***	***x_**4**_***	**X_**1**_**	**X_**2**_**	**X_**3**_**	**X_**4**_**	**Observed**	**Estimated (*Y*)**
1	−1	−1	−1	−1	12.5	1.5	2.5	60	0.465	1.952
2	1	−1	−1	−1	15.5	1.5	2.5	60	0.316	−1.447
3	−1	1	−1	−1	12.5	2.5	2.5	60	0.706	3.107
4	1	1	−1	−1	15.5	2.5	2.5	60	0.268	−0.292
5	−1	−1	1	−1	12.5	1.5	6.5	60	1.721	4.061
6	1	−1	1	−1	15.5	1.5	6.5	60	1.306	0.662
7	−1	1	1	−1	12.5	2.5	6.5	60	1.935	5.216
8	1	1	1	−1	15.5	2.5	6.5	60	1.451	1.817
9	−1	−1	−1	1	12.5	1.5	2.5	108	6.763	6.580
10	1	−1	−1	1	15.5	1.5	2.5	108	6.038	3.181
11	−1	1	−1	1	12.5	2.5	2.5	108	7.107	7.735
12	1	1	−1	1	15.5	2.5	2.5	108	5.440	4.336
13	−1	−1	1	1	12.5	1.5	6.5	108	17.431	15.360
14	1	−1	1	1	15.5	1.5	6.5	108	11.071	11.961
15	−1	1	1	1	12.5	2.5	6.5	108	15.709	16.515
16	1	1	1	1	15.5	2.5	6.5	108	12.478	13.116
17	−2	0	0	0	11	2	4.5	84	16.005	12.117
18	2	0	0	0	17	2	4.5	84	2.345	5.319
19	0	−2	0	0	14	1	4.5	84	1.081	2.630
20	0	2	0	0	14	3	4.5	84	8.019	4.940
21	0	0	−2	0	14	2	0.5	84	0.299	1.732
22	0	0	2	0	14	2	8.5	84	14.968	12.621
23	0	0	0	−2	14	2	4.5	36	0.001	−4.178
24	0	0	0	2	14	2	4.5	132	10.846	11.749
25	0	0	0	0	14	2	4.5	84	2.479	3.785
26	0	0	0	0	14	2	4.5	84	2.116	3.785
27	0	0	0	0	14	2	4.5	84	2.479	3.785
28	0	0	0	0	14	2	4.5	84	2.766	3.785
29	0	0	0	0	14	2	4.5	84	2.240	3.785
30	0	0	0	0	14	2	4.5	84	2.521	3.785

a *X_1_, beet molasses (%); X_2_, yeast extract (%); X_3_, inoculum size (OD_600_); X_4_, culture time (h)*.

b *Extracellular ScAGal activity (μmol.min.mL^−1^) to pH 4 and 40°C*.

**Figure 7:**
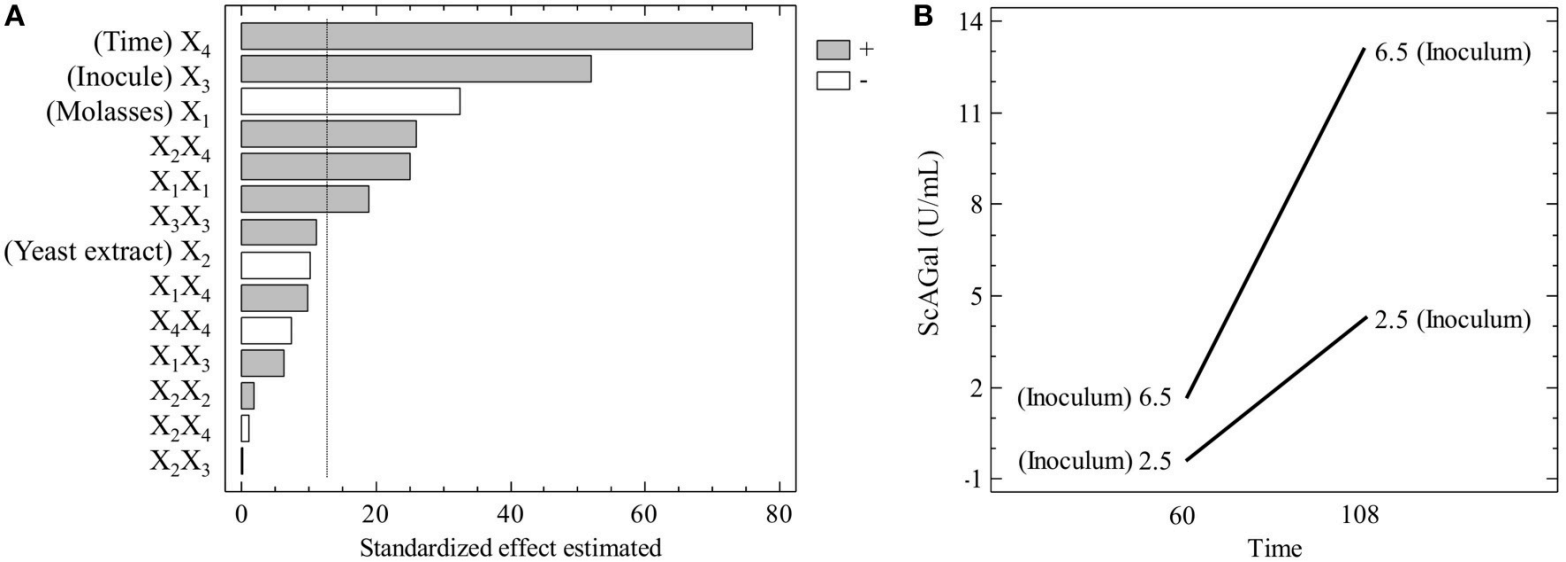
Pareto graphic and main interactions on the production of ScAGal. **(A)** Independent variables on the response at the 95% level of significance (vertical line) before remove the statistically insignificant effects. **(B)** Interaction Time/Inoculum effect, where the time varies from −1 to +1 while the inoculum remains constant at the value +1 (up line) and −1 (down line).

To validate the significance of the model obtained, the ANOVA presented in Table 4 was performed. The Pareto graphic analysis of the model shows the significance of the influence of the variables on the response (Figure 7A). The effects statistically not significant at the 95% confidence level were excluded from the model (and do not appear in Table 4), except yeast extract concentration that was the closest to the significance limit. Inoculum size and incubation time showed a positive effect on the response, whereas molasses concentration effect was negative. The effect of yeast extract concentration was smaller than the other three variables as corresponds to its lower significance level. Moreover, the representation of the single interaction that was significant, between inoculum size and incubation time (Figure 7B), shows that the increase of the inoculum size provokes a higher increase of the response if the incubation time also increases. The correlation coefficient (*R*^2^) that explains the 86.8% of the variation of the response and the *p*-value > 0.05 for the lack of fit test prove that the regression model is significant and can be used to describe the production of ScAGal into the defined experimental domain. Therefore, the following equation describes the relationship between response and variables: Extracellular alpha-galactosidase activity (*Y*) = 3.78–1.70*x*_1_+ 0.58*x*_2_ + 2.72*x*_3_ + 3.98*x*_4_ + 1.23*x*_1_ *x*_1_+ 0.85*x*_3_ *x*_3_ + 1.67C*x*_3_*x*_4_.

**Table 4:** ANOVA for the response surface quadratic model to the optimization of the ScAGal production by BJ3505Δ*suc2*/YEp*MEL1*^a^.

**Effects^**b**^**	**Sum of squares**	**Df^**c**^**	**Mean square**	**F-ratio**	***p*-value^**d**^**
*x_1_*	69.323	1	69.323	1052.18	0.0196
*x_2_*	8.003	1	8.003	121.47	0.0576
*x_3_*	177.872	1	177.872	2699.75	0.0123
*x_4_*	380.480	1	380.480	5774.96	0.0084
*x_1_x_1_*	37.071	1	37.071	562.66	0.0268
*x_3_x_3_*	17.520	1	17.520	265.92	0.0390
*x_3_x_4_*	44.499	1	44.499	675.41	0.0245
Lack of Fit	110.244	17	6.485	98.43	0.0780
Pure Error	0.065	1	0.065		
Total	835.492	25			

a *R^2^ = 86.80%; adjusted R^2^ = 81.66%; standard error = 0.256; mean absolute error = 1.74*.

b *Linear effects (x_1_, molasses; x_2_, yeast extract; x_3_, inoculum size; x_4_, culture time), quadratic effects (x_1_x_1_; x_3_x_3_), and interaction effect (x_3_x_4_)*.

c *Df, Degrees of freedom*.

d *p ≤ 0.05 denotes a statistically significant difference*.

**Figure 8:**
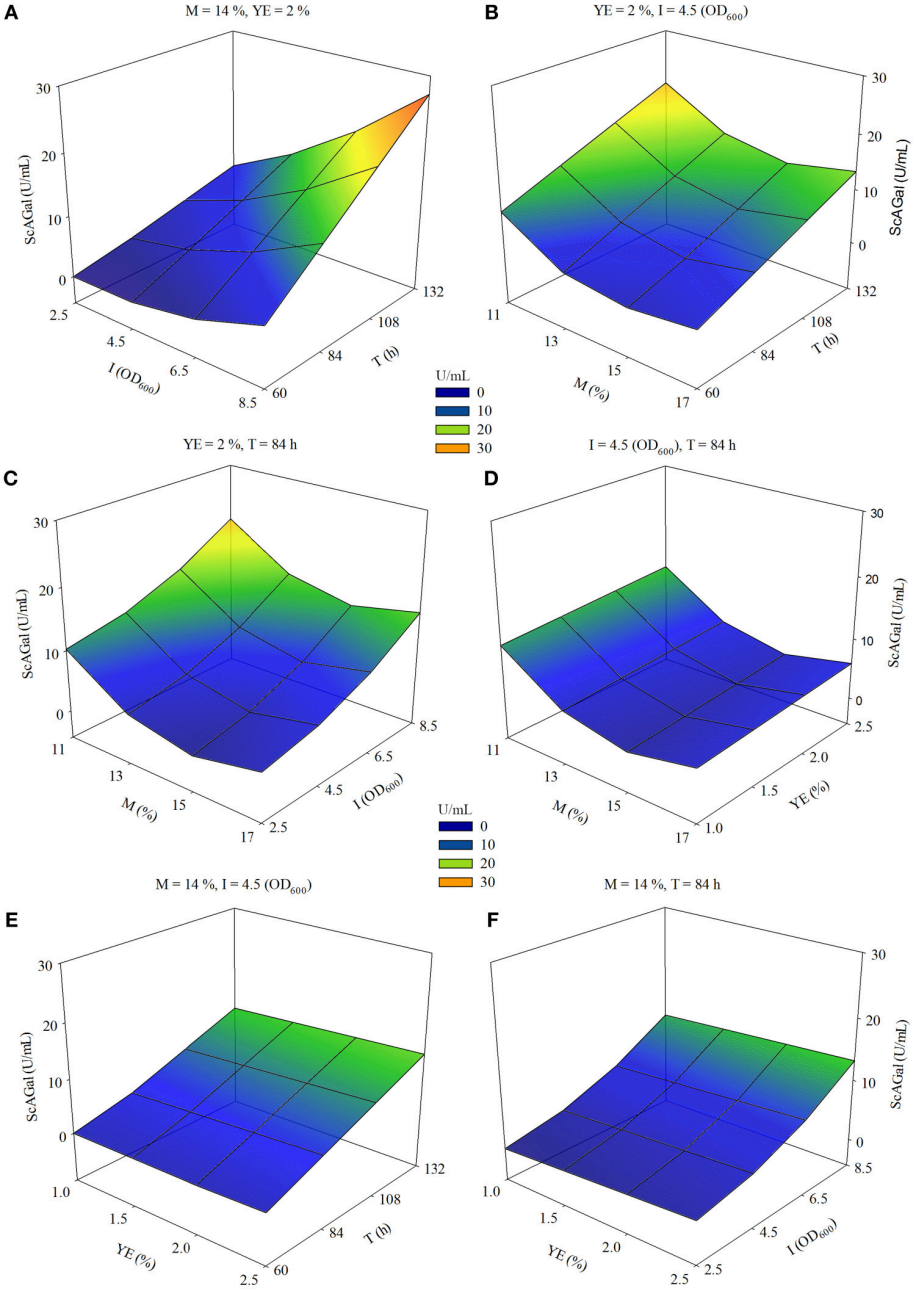
Response surface-contour plots for extracellular ScAGal production by the strain BJ3505Δ*suc2*/YEp*MEL1* on beet molasses based medium using RSM. Estimated extracellular alpha-galactosidase activity (ScAGal) as function of **(A)** inoculum size (I) and time (T); **(B)** beet molasses (M) and time (T); **(C)** beet molasses (M) and inoculum size (I); **(D)** beet molasses (M) and yeast extract (YE); **(E)** yeast extract (YE) and time (T); **(F)** yeast extract (YE) and inoculum size (I). The values of the third and fourth variables were remained constant at level 0.

SRM and contour plot analysis reinforce that the effect of increasing incubation time (T) is more positive as inoculum size (I) increases (Figure 8A), and also when molasses concentration (M) decreases (Figure 8B). The combination of maximum I and minimum M also drives to an increase in ScAGal production (Figure 8C), while the effect of yeast extract (YE) is scarcely relevant in the experimental domain studied (Figures 8D–F).

The maximum ScAGal production value is obtained in the experimental domain conditions *x*_1_ = −2, *x*_2_ = 0, *x*_3_ = +2, *x*_4_ = +2, which correspond to the real values 11% M, 2% YE, 8.5OD_600_ I, and 132 h T, respectively. According to the estimated model, an increase in YE (*x*_2_ = 0, *X*_2_ = 2%) would slightly increase the ScAGal production (above 4%) compared to the value *x*_2_ = −2 (*X*_2_ = 1%) of the same variable, keeping the rest of the conditions optimized. Therefore, we decided to use 1% YE to economize the productive process and since it is also the concentration usually used in commercial culture media. Under these conditions, the strain BJ3505Δ*suc2*/YEp*MEL1* yielded the maximum extracellular alpha-galactosidase activity of 24 U/mL (Figure S2). This is a value close to predicted by the model (34 U/mL) and represents an improvement of the 72% with respect to the ScAGal production observed in the first studies performed in this work.

Therefore, in addition to being sustainable and low-cost, we have proved that molasses are also promising substrates for ScAGal production with the BJ3505Δ*suc2*/YEp*MEL1* strain engineered in this work.

### Scale-Up to 2-L Bioreactor

**Figure 9:**
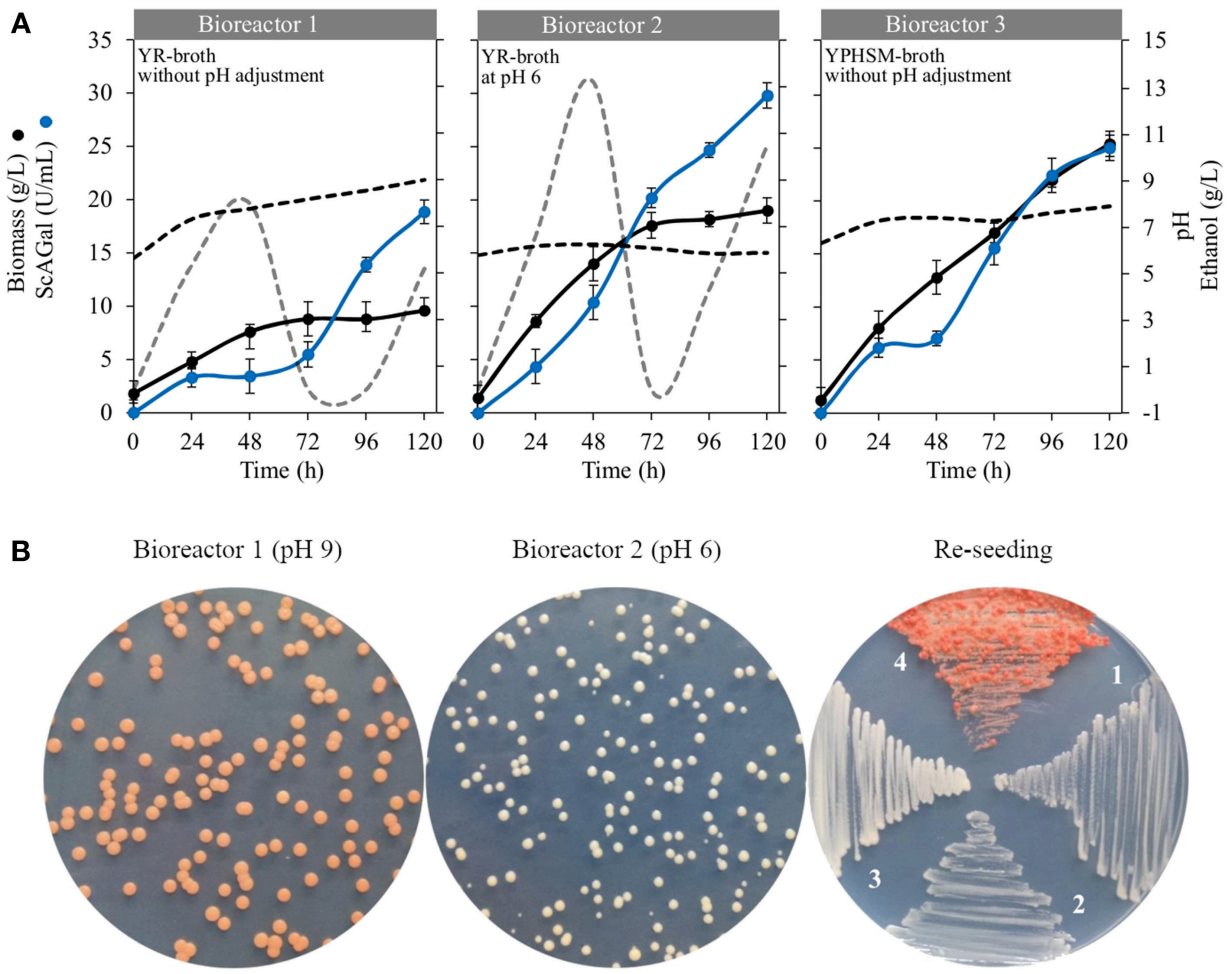
Cultivation of strain BJ3505Δ*suc2*/YEp*MEL1* in bioreactors under controlled pH conditions. **(A)** Time course of cellular biomass (black circles), extracellular alpha-galactosidase activity (blue circles), and pH (black dashed line) in optimized YR (Bioreactor 1 and 2) and YPHSM (Bioreactor 3). The production of ethanol (gray dashed line) is shown in bioreactors 1 and 2**. (B)** Macroscopic visualization of cell colonies from ended-bioreactors, at pH 9 and pH 6, and re-seeding on selective medium CM-Trp. Re-seeding: 1, BJ3505/YEp*MEL1* in YPHSM (pH 7); 2, BJ3505Δ*Suc2*/YEp*MEL1* in YPHSM (pH 7); 3, BJ3505Δ*Suc2*/YEp*MEL1* in YR (pH 6); 4, BJ3505Δ*Suc2*/YEp*MEL1* in YR (pH 9).

The strain BJ3505Δ*suc2*/YEp*MEL1* was cultured in 2-L bioreactors with 1-L working volume of optimized YR (beet molasses-yeast extract) medium under controlled pH conditions. *S. cerevisiae* strains grow well between pH 4.5 and 6.5 (Walker and Stewart, 2016), and we decided to maintain bioreactors at pH 6 since it slightly improved the ScAGal production according to our previously observed experiments (Álvarez-Cao, 2017). Figure 9A shows the monitoring of bioreactors using optimized YR without (Bioreactor 1) and with (Bioreactor 2) adjustments at pH 6 during the culture time, where we found unexpected results that had not been considered before. First, cellular growth stopped at 72 h in both cases, but biomass and extracellular alpha-galactosidase activity were approximately double when the culture was maintain at pH 6, reaching about 20 g/L and 30 U/L, respectively, at 120 h of culture. Second, the pH value suddenly increased to pH 9 when it was not adjusted during the course of the culture. Apart from ScAGal, other metabolites were produced depending on the culture phase, shown in Table 5. Thus, during the first 24 h, the strain used all the glucose and only 73% (Bioreactor 1) or 82% (Bioreactor 1) of the fructose to produce ethanol and glycerol as co-metabolites. At the same time, galactose appears in the medium coming from 5% (Bioreactor 1) to 25% (Bioreactor 2) of the hydrolysis of raffinose by the action of ScAGal. Galactose concentration was maintained constant thereafter in both cultures, meaning that the strain prefers to use other monosaccharides (Marques et al., 2015). On the other hand, in the bioreactor without adjustment at pH 6 (Bioreactor 1; Figure 9A), from 24 to 48 h the strain metabolism was preferetially fermentative producing 8 g/L ethanol and 1.6 g/L glycerol while ScAGal activity was kept constant. During the following 48–96 h, strain's metabolism shifted to respiratory using ethanol and glycerol as carbon sources, and ScAGal expression sharply increased. The next 24 h, the production of ethanol and glycerol re-started and ScAGal activity reached 20 U/mL at 120 h of culture. However, when the bioreactor pH was maintained at 6 (Bioreactor 2; Figure 9A), the strain metabolism was respiro-fermentative during all culture, producing ethanol, glycerol, and ScAGal until 48 h, from 48 to 72 h consumed all the previously produced ethanol and a part of the glycerol while ScAGal increased up to 20 U/mL, and from 72 to 120 h, ethanol was newly produced and levels of ScAGal reached the highest values obtained (30 U/mL). If both cultures were extended, ethanol consumption and ScAGal production could be increased. These results are superior to those obtained in a similar characteristics bioreactor using the rich and much more expensive medium YPHSM with 5% (w/v) glucose (a level of assimilable sugars comparable to YR medium) that yielded more biomass (25 g/L) but less ScAGal (25 U/mL) (Bioreactor 3; Figure 9A). So, scale-up to 1-L culture in bioreactor with pH control allowed to increase a 21% the levels of ScAGal production compared with the best results obtained using shaken flasks (Figure S2).

**Table 5:** HPLC analysis to quantify the conversion of substrates to products during the cultivation of bioreactors with strain BJ3505Δ*suc2*/YEp*MEL1*^a^.

**g/L**	**YR**	**Bioreactor 1**^**b**^					**Bioreactor 2**^**c**^				
		**24 h**	**48 h**	**72 h**	**96 h**	**120 h**	**24 h**	**48 h**	**72 h**	**96 h**	**120 h**
Raf	4.55	4.35	4.29	4.35	4.42	4.29	3.40	3.35	3.40	3.45	3.35
Suc	92.65	91.12	106.28	106.56	113.69	100.55	93.75	87.90	87.30	84.90	61.30
Glu	1.30	0.00	0.00	0.00	0.00	0.00	0.00	0.05	0.00	0.00	0.00
Gal	0.50	0.82	0.91	0.86	0.89	0.82	1.30	1.30	1.35	1.30	1.25
Fru	3.55	0.64	0.82	0.56	0.65	2.06	0.95	0.55	0.40	0.60	0.85
Gly	0.45	0.98	1.62	0.80	0.27	1.00	1.30	1.05	0.40	0.70	2.65
Eth	0.00	5.34	7.98	0.00	0.00	5.19	6.60	13.20	0.00	4.30	10.50
Sor	–	1.00	1.00	1.00	1.00	1.00	1.00	1.00	1.00	1.00	1.00

a *Concentrations from 4 to 0.06 mg/mL of a mixture of raffinose (Raf), sucrose (Suc), galactose (Gal), glucose (Glu), fructose (Fru), glycerol (Gly), and ethanol (Eth) were used as external standard. Both samples and external standard were added with 1 mg/mL sorbitol (Sor) used as an internal standard, N = 3 ± DE*.

b *Bioreactor 1 was carried out without adjusted pH during the culture course*.

c *Bioreactor 2 was maintained at pH 6 during the culture course*.

Strikingly, when cells coming from ended-cultures of both bioreactors, at pH 9 and pH 6, were seeded in solid CM-Trp medium, only those coming from pH 9 acquired pink color (Bioreactor 1; Figure 9B) while the second ones maintained the normal phenotype (Bioreactor 2; Figure 9B). This characteristic was maintained during successive re-seedings for 10 months (Re-seeding; Figure 9B). Moreover, pink colonies grew faster than white ones and with a peculiar morphology. On the other hand, plasmid stability resulted to be about 98% in both bioreactors, independently of pH value, after 120 h of culture.

## Discussion

Molasses are highly abundant sub-products of the food industry that are hitherto being fermented to ethanol at industrial level due to their richness in sucrose. The yeasts employed are adapted strains of *S. cerevisiae*, that produces high amounts of extracellular invertase, a native enzyme that hydrolyses sucrose into glucose and fructose, and is a Crabtree positive yeast, which means that preferentially ferments high concentrations of sugars to ethanol even under aerated conditions (Marques et al., 2015).

In this work, we started from the hypothesis that a mutant strain not-producing invertase (Δ*suc2*), i.e., unable to use sucrose, would metabolize the sugars present in molasses at lower concentration (raffinose, glucose, fructose) that could be used to produce a recombinant protein. In our case, the selected recombinant protein was ScAGal, an enzyme widely used in several industries and with large market, that would also simultaneously allow to use the raffinose present in molasses since ScAGal shows a high affinity for this sugar (Fernández-Leiro et al., 2010). The remaining sucrose could be fermented by wild type *S. cerevisae* in a coupled second-step classical industrial process.

**Figure 10:**
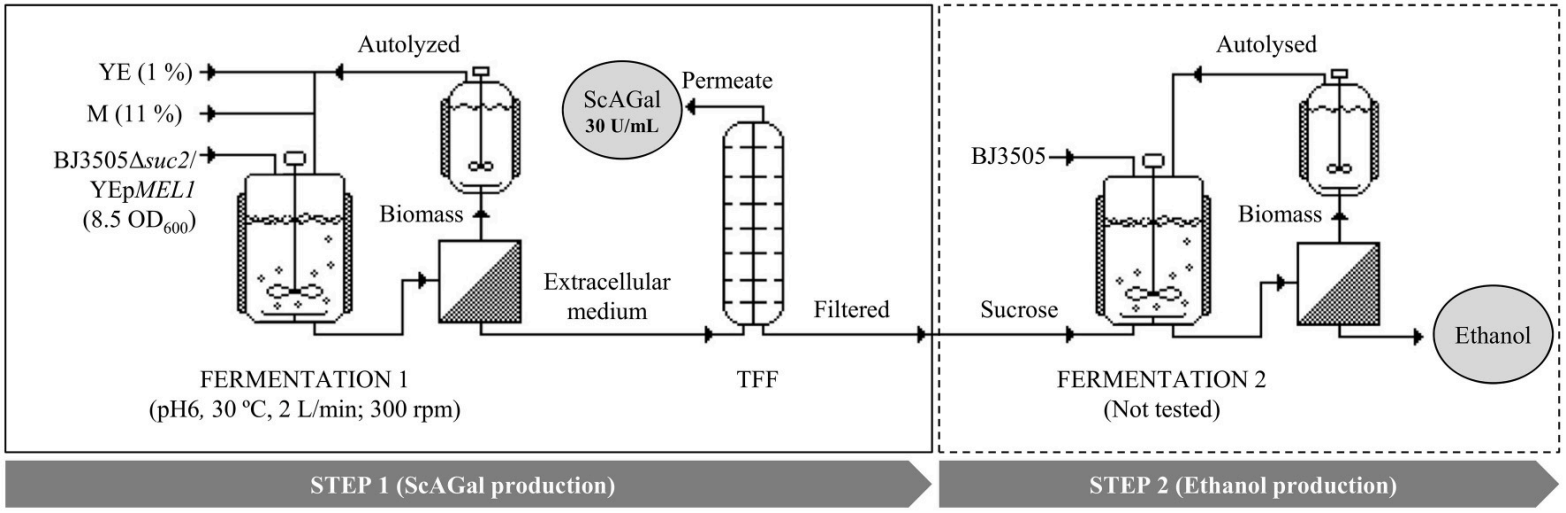
Simulation of a two-step bioprocess to obtain ScAGaL and ethanol from beet molasses-based medium. The ended-culture of the fermentation 1 carried out by the invertase-deficient strain BJ3505Δ*suc2*/YEp*MEL1* is centrifuged, and the extracellular medium filtered by TTF to obtain a permeate with the recombinant protein, ScAGal (Step 1). The sucrose recovered in the filtrate is used in the fermentation 2 to produce ethanol by a strain of *S. cerevisiae* with invertase activity (Step 2). The biomass recovered from both fermentations can be used as yeast extract (YE) supplement after an autolysis treatment. M, beet molasses; YE, yeast extract; TFF, tangential flow filtration. Modificated imagen from SuperPro Designer.

Figure 10 shows the scheme of a proposed productive process of ScAGal and bioethanol in the conditions optimized in this work, using beet molasses as substrate and the constructed engineered yeast strain. In the first step, ScAGal would be obtained from cultures of the strain BJ3505Δ*suc2*/YEp*MEL1* in molasses medium. In the second step, bioethanol would be obtained from cultures of any wild type yeast strain, in our case the strain of origin BJ3505, in post-incubate cell-free sucrose-rich medium resulting from the first step after ScAGal recovery by tangential flow filtration (TFF). The ethanol produced in step 2 could be extracted for biofuel use, and also recycled as carbon source for ScAGal production in the step 1. Moreover, one part of the yeast biomass obtained (of each of the two strains) would serve as inoculum of the following culture, and the rest would be autolyzed and then used to supplement molasses instead of the more expensive commercial yeast extract.

During the research conducted to develop the first step of this proposed coupled process, we found several interesting observations. On the one hand, the Δ*suc2* strain produced more extracellular ScAGal from molasses than the corresponding wild type strain. We attributed this result to the relief of the secretory route due to the absence of invertase (Liljeström et al., 1991) and to the shift to a respiratory metabolism due to the inability to use sucrose (Finn et al., 2006). In addition, ScAGal became easier to purify from the culture medium without contaminating invertase. On the other hand, the Δ*suc2* strain presented some curious morphological changes. In the first place, the absence of vacuolar particles that are observed in the wild type strain growing in molasses, and that in the case of the mutant are cytosolic. Under stress conditions, the vacuole is prone to flow between non-fragmented and fragmented forms due in part to its association with other cellular organelles (endoplasmic reticulum, Golgi complex, secretory, and endocytic vesicles) as components of interconnected branched systems (Gibson et al., 2007). This change of morphology could be attributed to a stress response due to environmental factors, such as glucose depletion, tolerance to ethanol (Gibson et al., 2007; Kato et al., 2011; Grousl et al., 2013), or to the switch from fermentative to respiratory metabolism since the wild type strain growing in ethanol does not present such vacuolar particles. In the second place, the Δ*suc2* strain gains a pink color in a pH-dependent manner that could be related to the synthesis of carotenoid pigments by yeast as has also been reported in the literature for other microorganisms (Querijero-Palacpac et al., 1990; Schmidt et al., 2011). However, the molecular mechanism of these morphological changes merits to be the subject of further research.

It is well-known that high concentrations of sucrose exert hyperosmotic stress on *S. cerevisiae* which synthesizes antioxidant and osmoregulatory molecules to avoid lethal injuries, such as trehalose and glycerol, among others (Gibson et al., 2007). The accumulation of trehalose, as osmotic protector, with similar retention time than sucrose or the induction of the synthesis of other hydrolases involved in sucrose metabolism (Marques et al., 2015) could explain the increase and decrease in sucrose levels observed during cultures without and with pH control, respectively (Table 5).

Finally, we reported for the first time the statistical optimization of the production of ScAGal from molasses (using CCD) with high yields of enzyme production, while the fermentation to ethanol of the remaining sucrose was just verified but not optimized since this procedure is nowadays ongoing at industrial scale. In addition, the stability of the plasmid expressing ScAGal is not a problem since 98% of the cells growing in molasses retained the plasmid at the end of the cultures. In fact, we obtained a production of 30 U/mL of ScAGal which is 1.6-fold higher than the recently reported with a *Kluyveromyces lactis* engineered strain growing in a mixture of cheese whey and molasses (Álvarez-Cao et al., 2018) and higher than the 10.4 U/mL obtained for *Aspergillus fumigatus* alpha-galactosidase expressed in *A. sojae* in an optimized synthetic medium in the presence of 10.5% (w/v) molasses (Gurkok et al., 2011). The alpha-galactosidase yield reached is among the highest of those obtained in submerged fermentation for the alpha-galactosidase enzyme of different origins on various carbon sources (Anisha, 2017).

## Conclusion

A procedure for valuation of beet molasses has been developed that consists in the high-yield production of ScAGal, an enzyme with wide market, by an engineered yeast strain from the less abundant sugars, followed by ethanol fermentation of sucrose, the most abundant sugar, by a wild type strain. The engineered strain is a Δ*suc2* mutant transformed with a plasmid that drives secretion of ScAGal to the culture medium. The mutation causes a shift to a more respiratory metabolism in molasses and facilitates extracellular ScAGal purification by avoiding contamination with invertase.

## Data Availability

All datasets generated for this study are included in the manuscript and/or the supplementary files.

## Author Contributions

M-EC, M-IG-S, and MB contributed conception and design of the study. M-EA-C performed the experimental work (data acquisition); M-EA-C, M-IG-S, and MB contributed to analysis or interpretation of data. M-EA-C and M-IG-S wrote the first draft of the manuscript. All authors contributed to manuscript revision, read, and approved the submitted version.

### Conflict of Interest Statement

The authors declare that the research was conducted in the absence of any commercial or financial relationships that could be construed as a potential conflict of interest.
