# Supplementary material for: Bioconversion of Beet Molasses to Alpha-Galactosidase and Ethanol
Source: Front Microbiol. 2019 Mar 7;10:405. doi: 10.3389/fmicb.2019.00405 (PMC6416216; doi:10.3389/fmicb.2019.00405)
Supplement: Supplementary file 1 [file Data_Sheet_1.docx]

Supplementary Material

**Bioconversion of Beet Molasses to Alpha-Galactosidase and Ethanol**

**María-Efigenia Álvarez-Cao^1^, María-Esperanza Cerdán^1^, María-Isabel González-Siso^1^*, Manuel Becerra^1^**

*** Correspondence:**

Corresponding Author: [migs@udc.es](mailto:migs@udc.es)

**Table S1.** Oligonucleotides used in this study.

| Primer | Sequence (5´ to 3´) ^a^ | Strand ^b^ | Applied strategy |
| --- | --- | --- | --- |
| P1 | CCACGATGGTAATAGACG | F | *SUC2* cassette |
| P2 | GAGATAATATCCTCCAGTCC | R | *SUC2* cassette |
| P3 | GCGGATCCCCTTTAGCTGTTCTATATGCTGC | F | *Bgl*II site; *URA3* cassette |
| P4 | CCGGTACCCCACCTGACGTCTAAGAAACC | R | *Kpn*I site; *URA3* cassette |
| P5 | CGCTGTATTCTTAGGTGCTATACC | F | Upstream integration |
| P6 | CATTTTCAATATTTCTAACTCACTAACC | R | Upstream integration |
| P7 | TTGTACTTGGCGGATAATGC | F | Downstream integration |
| P8 | ACCAATCGTAACCTTCATCTCTT | R | Downstream integration |

^a^ Engineered restriction sites are underlined. ^b^ F, forward strand; R, reverse strand.

**Table S2.** Analysis of variance ^a^ of ScAGal expression systems using YPHSM as culture medium.

| STATISTICAL SUMMARY | | | | | | |
| --- | --- | --- | --- | --- | --- | --- |
| Expression systems (Groups) | | Mean | Minimum | Maximum | Bias | Curtosis |
| BJ3505∆*suc2* | YEp*MEL1* (1) | 35.33 | 23.30 | 43.85 | -0.685 | -0.811 |
|  | YEp*MEL1*His (2) | 20.75 | 12.66 | 25.49 | -0.985 | -0.301 |
| BJ3505 | YEp*MEL1* (3) | 33.94 | 23.36 | 41.70 | -0.678 | -0.749 |
|  | YEp*MEL1*His (4) | 21.26 | 14.85 | 26.57 | -0.300 | -0.141 |
| ANOVA | | | | | | |
| Source of variation |  | SS | DF | MS | F*-*value | *p*-value |
| Between-Groups |  | 1493.92 | 3 | 497.97 | 12.43 | 0.000 |
| Within-Groups |  | 1121.86 | 28 | 40.07 |  |  |
| Total (Corrected) |  | 2615.78 | 31 |  |  |  |
| MULTIPLE RANGE TEST ^b^ | | | | | | |
| Contrast | Significant ^c^ | Different | +/- Limits |  |  |  |
| 1 - 2 | Yes | 14.576 | 6,483 |  |  |  |
| 1 - 3 | No | 1.392 | 6,483 |  |  |  |
| 1 - 4 | Yes | 14.066 | 6,483 |  |  |  |
| 2 - 3 | Yes | -13.184 | 6,483 |  |  |  |
| 2 - 4 | No | -0.510 | 6,483 |  |  |  |
| 3 - 4 | Yes | 12.674 | 6,483 |  |  |  |

^a^ The extracellular alpha-galactosidase activity of the cultures tested is the independent variable or study response. ^b^ Fisher's Least Significant Difference test was used to discriminate between the means. ^c^ Denotes a statistically significant difference between pairs of experimental groups (contrast). SS, sum of square; DF, degrees of freedom; MS, mean square.


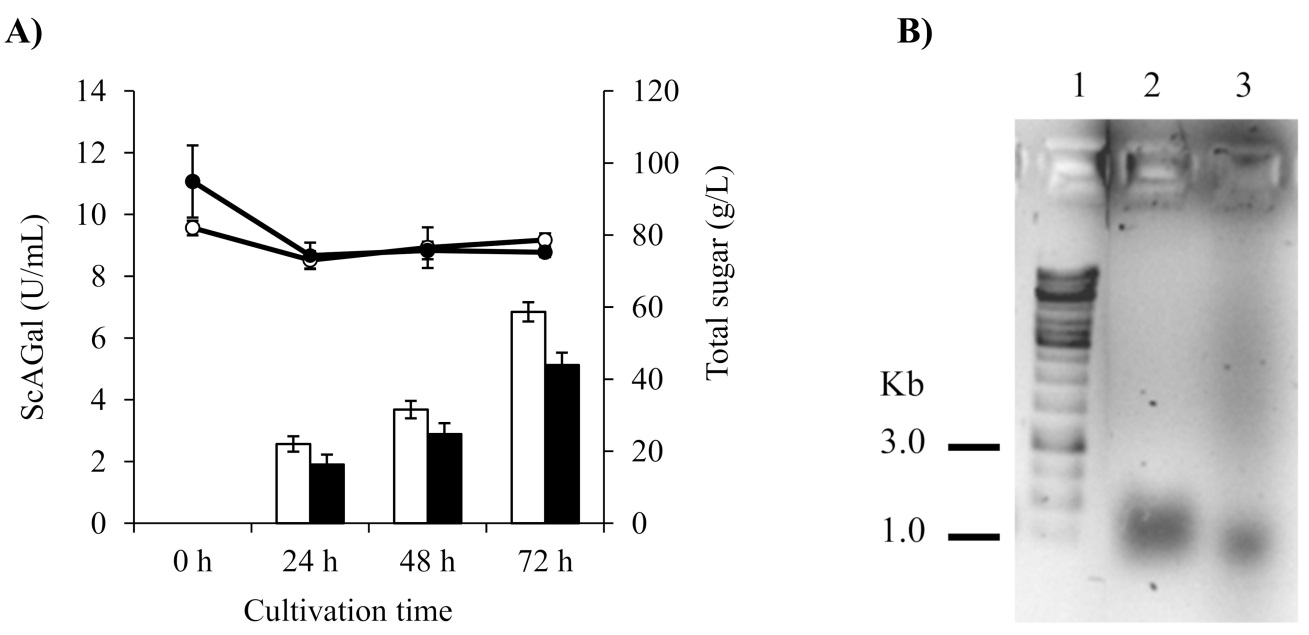


**Figure 1S.** Recycling of biomass of the recombinant strain BJ3505Δ*suc2*/YEp*MEL1* as molasses supplement. **(A)** Extracellular alpha-galactosidase activity (bar plot) and residual sugar content (line plot) of cultures in beet molasses supplemented with commercial yeast extract (empty symbols) or biomass autolyzed (full symbols) as source of nitrogen. The culture media were inoculated with an initial OD_600_ of 4. N = 3 ± SD. YR, 8 % beet molasses and 1 % commercial yeast extract; YRaut, 8 % beet molasses and 0.5 % autolyzed biomass. **(B)** Comparative analysis of autolyzed and commercial yeast extracts by electrophoresis in 1.5 % agarose gel. Lane 1, GeneRuler 1Kb DNA Ladder (Thermo Scientific); lane2, autolyzed biomass according to methodology (Section 2.4); lane 3, 1 % commercial yeast extract.


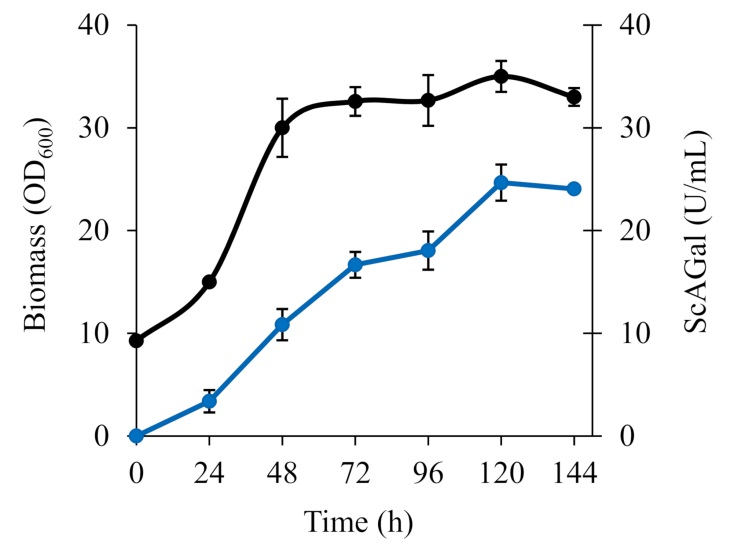


**Figure 2S.** Production of ScAGal by BJ3505Δ*suc2*/YEp*MEL1* using optimized beet molasses medium. Biomass (black circles) and extracellular alpha-galactosidase activity (blue circles). The culture media were performed with 11% molasses, 1% yeast extract, an inoculum size of OD_600_ = 8.5 and maintaining the other conditions described (Section 2.4.). N = 3 ± SD.
